# DNA Methylation Perturbations in Genes Involved in Polyunsaturated Fatty Acid Biosynthesis Associated with Depression and Suicide Risk

**DOI:** 10.3389/fneur.2015.00092

**Published:** 2015-04-28

**Authors:** Fatemeh Haghighi, Hanga Galfalvy, Sean Chen, Yung-yu Huang, Thomas B. Cooper, Ainsley K. Burke, Maria A. Oquendo, J. John Mann, M. Elizabeth Sublette

**Affiliations:** ^1^Department of Psychiatry, James J. Peters Veterans Affairs Medical Center, New York, NY, USA; ^2^Fishberg Department of Neuroscience, Icahn School of Medicine at Mount Sinai, New York, NY, USA; ^3^Friedman Brain Institute, Icahn School of Medicine at Mount Sinai, New York, NY, USA; ^4^Department of Psychiatry, Columbia University College of Physicians and Surgeons, New York, NY, USA; ^5^Division of Biostatistics, New York State Psychiatric Institute, New York, NY, USA; ^6^Division of Molecular Imaging and Neuropathology, New York State Psychiatric Institute, New York, NY, USA; ^7^Nathan S. Kline Institute for Psychiatric Research, Orangeburg, NY, USA; ^8^Department of Radiology, Columbia University College of Physicians and Surgeons, New York, NY, USA

**Keywords:** DNA methylation, epigenetics, suicide, major depressive disorder, polyunsaturated omega-3/omega-6 fatty acids, Fads1 fatty acid desaturase, elongation of very long-chain fatty acids protein 5

## Abstract

Polyunsaturated fatty acid (PUFA) status has been associated with neuropsychiatric disorders, including depression and risk of suicide. Long-chain PUFAs (LC-PUFAs) are obtained in the diet or produced by sequential desaturation and elongation of shorter-chain precursor fatty acids linoleic acid (LA, 18:2*n*-6) and α-linolenic acid (ALA, 18:3*n*-3). We compared DNA methylation patterns in genes involved in LC-PUFA biosynthesis in major depressive disorder (MDD) with (*n* = 22) and without (*n* = 39) history of suicide attempt, and age- and sex-matched healthy volunteers (*n* = 59). Plasma levels of selected PUFAs along the LC-PUFA biosynthesis pathway were determined by transesterification and gas chromatography. CpG methylation levels for the main human LC-PUFA biosynthetic genes, fatty acid desaturases 1 (*Fads1*) and 2 (*Fads2*), and elongation of very long-chain fatty acids protein 5 (*Elovl5*), were assayed by bisulfite pyrosequencing. Associations between PUFA levels and diagnosis or suicide attempt status did not survive correction for multiple testing. However, MDD diagnosis and suicide attempts were significantly associated with DNA methylation in *Elovl5* gene regulatory regions. Also the relative roles of PUFA levels and DNA methylation with respect to diagnostic and suicide attempt status were determined by least absolute shrinkage and selection operator logistic regression analyses. We found that PUFA associations with suicide attempt status were explained by effects of *Elovl5* DNA methylation within the regulatory regions. The observed link between plasma PUFA levels, DNA methylation, and suicide risk may have implications for modulation of disease-associated epigenetic marks by nutritional intervention.

## Introduction

**Figure 1:**
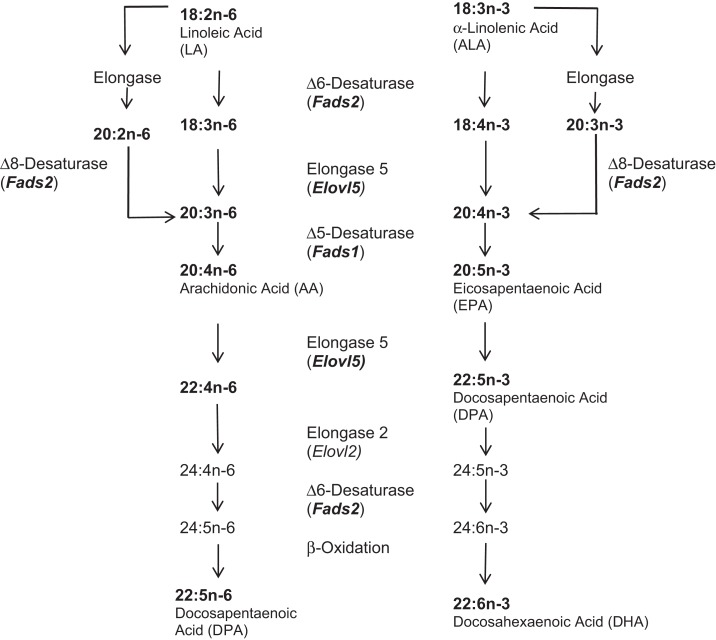
**LC-PUFA biosynthesis pathway {adapted from Refs. (6, 7)}**. PUFA species and biosynthetic genes included in these analyses are indicated in bold.

Imbalances in polyunsaturated fatty acids (PUFAs) may contribute to psychiatric illness, including mood disorders and suicidal behavior (1). PUFAs are not endogenously produced in humans and thus must be derived from diet. Flax seeds, eggs from *n*-3 (omega-3) PUFA-fed chickens, and oily fish – including salmon, herring, and sardines, tend to contain the highest levels of *n-*3 PUFAs compared to other sources. Although intake is undoubtedly a major determinant of PUFA effects since long-chain (LC)-PUFAs cannot be manufactured *de novo* in mammals, additional physiological factors affecting PUFA bioavailability may also mediate the clinical effects of low *n*-3 PUFA levels. Relevant here is the sequential desaturation and elongation pathway that produces LC-PUFAs from shorter-chain precursor PUFAs linoleic acid (LA, 18:2*n*-6) and α-linolenic acid (ALA, 18:3*n*-3) (Figure 1). The principal enzymatic reactions involved are Δ6-desaturation (2) and Δ8-desaturation (3) catalyzed by the fatty acid desaturase 2 (*Fads2)* gene product; Δ5-desaturation catalyzed by the fatty acid desaturase 1 (*Fads1)* gene product (4); and elongase reactions catalyzed by the elongation of very long-chain fatty acids proteins (*Elovl5* and *Elovl2)* gene products (5) (see Figure 1).

*n*-3 PUFAs are critical to brain development, mental and neurological health, and cognitive functioning. In brain, docosahexaenoic acid (DHA, 22:6*n*-3), the end product of the *n*-3 PUFA biosynthetic pathway, tends to accrue in growth cones, synaptosomes, astrocytes, myelin, as well as microsomal and mitochondrial membranes (8). *n*-3 PUFAs are also involved in synaptogenesis and neurite outgrowth (9) as well as neurotransmitter signaling. Specifically, proper dopaminergic and serotonergic functioning – pathways implicated in mood disorders and suicidal behavior – rely on *n*-3 fatty acids (10). The potential importance of omega-3 PUFAs to major depression has been demonstrated in a number of observational studies (11) and randomized controlled trials, with *n*-3 PUFAs showing effect sizes comparable to antidepressants (12, 13), and equivalent therapeutic effects to fluoxetine in a comparative study (14).

A number of studies have suggested a role for low *n*-3 PUFA intake in suicidal behavior. Suicide risk (15) and, in women, suicidal ideation (16) may be reduced in those who consume larger quantities of fish. *n*-3 PUFA levels are very low in suicide completers (17) and lower in attempters retrospectively (18) compared to depressed non-attempters, and we (19) found that low levels of DHA in major depressive disorder (MDD) patients predicted suicide attempt within 2 years. No differences in brain *n*-3 PUFA levels were detected in initial postmortem studies of adult and adolescent suicides compared with non-psychiatric controls (20–22), but differences in DHA levels emerged when depressed suicide completers were compared with controls without the confound of cardiovascular disease, also associated with low DHA (23).

Although not in the context of suicidal or related psychopathology, the role of diet in inducing chromatin and gene expression changes has been investigated by studying PUFA intake and expression of related biosynthetic enzymes. Studies in animals (7, 24–26) found that experimental changes in fat intake induce significant alterations in expression and activity of enzymes involved in PUFA biosynthesis, which can be mediated by DNA methylation status. For example, in a mouse model, methylation of *Fads2* promoter and intron 1 is increased in mothers and offspring when the diet is enriched in the *n*-3 PUFA ALA during pregnancy (26). DNA methylation, which occurs on the carbon-5 position of cytosines predominantly within CpG dinucleotides, is typically associated with repression of gene expression. For instance, in rats, high maternal fat intake during pregnancy and lactation increases *Fads2* promoter methylation status and decreases *Fads2* expression (25), as well as altering PUFA concentrations in liver phosphatidylcholine (PC) (25), effects that are reversed by switching to a soybean oil diet (25). In humans too, supplementation with *n*-3 PUFA induces changes in *Fads2* and *Elovl5* methylation status inverse to transcription rates (27).

In this study, we hypothesized that DNA methylation patterns in genes involved in PUFA biosynthesis may correlate with *n*-3 PUFA levels differentially in MDD compared with controls, and within MDD, in suicide attempters compared with non-attempters. We assayed CpG methylation levels in *Fads1*, *Fads2*, and *Elovl5* genes, examining methylation patterns within cytosine-rich CpG islands associated with *Fads1* and *Fads2*, as well as upstream and downstream regions proximal to the islands (denoted as CpG island shores). These shores tend to show tissue- and species-specific patterns of DNA methylation (28, 29). In contrast to hypomethylated CpG islands that tend to be highly conserved, the shore regions are ideally suited for targeted assessment of DNA methylation variation in clinical samples as used in the present study.

## Materials and Methods

### Samples

Written informed consent was obtained from adult participants (*n* = 120) in mood disorder studies approved by the New York State Psychiatric Institute’s Institutional Review Board. Participants included MDD patients (*n* = 61) and healthy control volunteers (*n* = 59), diagnosed using the Structured Clinical Interview for DSM-IV-TR Axis I Disorders (SCID-I) (30) and the SCID-I non-patient version (30), respectively (Table 1). At study entry, MDD patients had a depression severity score of at least 16 on the 17-item Hamilton Depression Rating Scale (31). We obtained a detailed history of each suicide attempt, defined as a self-destructive act with some intent to die. Determination of suicide attempts was made by consensus of expert research clinicians (inter-rater reliability coefficient: 0.97) using the Columbia Suicide History Form (32) according to criteria from the Columbia-Classification Algorithm for Suicide Attempts, based on nomenclature proposed by O’Carroll et al. (33). Participants were excluded if they had a history of neurological illness, active medical disease, or substance dependence. Depressed participants were required to be of antidepressant medications for at least 14 days (6 weeks for fluoxetine) prior to assessments to limit the potential confounding affect of these medications on PUFAs.

**Table 1:** **Demographics of sample participants by group**.

	Controls (*n* = 59)	MDD total (*n* = 61)	*t/X^2^*	*p*	MDD non-attempters (*n* = 39)	MDD suicide attempters (*n* = 22)	*t/X^2^*	*p*
**Age** Mean (SD)	36.9 (13.33)	35.1 (11.84)	0.8	0.452	36.6 (11.73)	32.6 (11.87)	1.3	0.211
**Male**	25 (42%)	29 (48%)	0.3	0.569	19 (49%)	10 (45%)	0.1	0.806
**Ethnicity**			2.8	0.094			0.3	0.605
Hispanic	9 (15%)	17 (28%)			10 (26%)	7 (32%)	
Non-Hispanic	50 (85%)	44 (72%)			29 (74%)	15 (68%)	
**Race**			5.6	**0.038**			0.1	0.932
Caucasian	28 (47%)	42 (69%)			27 (69%)	15 (68%)	
Non-Caucasian	31 (53%)	19 (31%)			12 (31%)	7 (32%)	

*Bold text indicates significance*.

### Determination of PUFA levels

Plasma PUFAs were purified as previously described (34). Briefly, direct transesterification (35) produced fatty acid methyl esters (FAMEs), which were extracted with hexane and subjected to gas chromatography according to a standardized procedure with highly consistent retention times. A representative sample of PUFA species along the biosynthetic pathway (see Figure 1) was chosen for quantification, and product/precursor ratios between consecutive steps along the pathway were calculated as surrogate indices of desaturase and elongase activity.

### DNA methylation assay via bisulfite sequencing

We targeted regions proximal to the transcription start site (TSS) of *Fads1*, *Fads2*, and *Elovl5* genes for methylation pyrosequencing (Table S1 in Supplementary Material). DNA was isolated from buffy coat via column purification using the QIAamp DNA Blood Mini Kit (Qiagen, Valencia, CA, USA), following the manufacturer’s protocol. DNA quantity was measured using a Qubit 2.0 Fluorometer and the Qubit dsDNA BR Kit (Life Technologies). DNA was then bisulfite treated by the EpiTect Bisulfite Kit (Qiagen, Valencia, CA, USA) using the manufacturer’s protocol. Methylation primers for the regions of interest were designed using Pyromark Assay Design Software (version 2.01.15) included with the Pyromark Q96 MD (Qiagen, Valencia, CA, USA), and PCR was performed on 10 μmol of both the forward and reverse primers using the *Taq* DNA Polymerase kit (Qiagen, Valencia, CA, USA). End-point PCR was performed for 45 cycles in a Bio-Rad MyCycler (Hercules, CA, USA) to ensure that all the primers were consumed, with the following cycling parameters used: 15 min at 95°C, 58°C for 30 s, 50°C for 30 s, and 95°C for 30 s. The primers were synthesized by Integrated DNA Technologies (Coralville, IA, USA), and the reverse primer from each set was biotinylated at the 5′ end. Primers used and the regions targeted are described in Table S1 in Supplementary Material.

Primer specificity for detecting methylated, bisulfite converted DNA was evaluated using 0–100% methylated, bisulfite converted DNA in 25% steps using the reagents provided by the EpiTect Control DNA kit (Qiagen, Valencia, CA, USA), with all primers used having expected linear standard curves. Methylation pyrosequencing was performed using a Pyromark Q96 MD, using the Pyromark Gold Q96 CDT Reagents following manufacturer’s instructions (both Qiagen, Valencia, CA, USA). Five microliters of amplified DNA product was mixed with 2 μl Streptavidin Sepharose beads (GE Healthcare, Rahway, NJ, USA), 40 μl binding buffer (Qiagen, Valencia, CA, USA), and 33 μl of nuclease free water (Life Technologies, Grand Island, NY, USA), followed by shaking on a P2 Orbit Digital Shaker (Labnet, Edison, NJ, USA) at 1400 rpm for 10 min. The immobilized biotinylated PCR product–Streptavidin Sepharose bead complex was captured using the Vacuum Prep Workstation (Qiagen, Valencia, CA, USA), and single strand purification was achieved by washing the vacuum prep tool sequentially with 75% ethanol, 0.2 M NaOH, and washing buffer (Qiagen, Valencia, CA, USA) for 30 s each. The unbiotinylated strand was dissociated and discarded, and the biotinylated strands were released to a 96-well microtiter plate, which contained 10.8 μl of annealing buffer (Qiagen, Valencia, CA, USA) and 1.2 μl complementary sequencing primer in each well. The plate was incubated at 80°C for 2 min, followed by slow cooling to room temperature. The processed mixture was loaded onto the PyroMark MD system equipped with PyroMark MD software for pyrosequencing, and the resulting pyrograms and associated sequences were generated and analyzed automatically using the PyroMark MD software.

### Statistical analysis

Methylation levels were averaged by region for each gene (*Fads1*, *Fads2*, and *Elovl5)*, using 5% trimming to avoid outlier sites within subject that could skew the average value. To mitigate the presence of outliers and skewed distribution, log-transformed PUFA and methylation values were used in all group comparisons. Separate ANCOVA models were employed to test for diagnostic group differences in PUFA and methylation levels, adjusted for age and sex. Two comparisons were run: (1) all MDD patients vs. healthy controls and (2) MDD attempters vs. MDD non-attempters. Significance levels were adjusted for multiple testing using the Benjamin–Hochberg linear step-down method (36), to preserve an experiment-wise false discovery rate of 5%. Significant models for MDD were adjusted for ethnicity and race. Next, all biological measures (and age and sex) were tested simultaneously as predictors of diagnosis and attempt. There were too many predictors compared to the group sample size, so, being cognizant of the shortcoming of stepwise variable selection methods, we used a feature selection technique that employs cross-validation to select the most important features. We performed model selection using the least absolute shrinkage and selection operator (LASSO) (37, 38) function cv.glmnet in the statistical language R, starting from the full set of PUFA and methylation variables (including age, sex, ethnicity, and race), with no pre-screening, and using cross-validation to select the optimal model. Since the LASSO logistic model does not currently provide a way to calculate significance levels, the variables in the final model were used as predictors in a logistic regression and these results are also provided for comparison.

## Results

For this study, we enrolled healthy volunteers/controls (*n* = 59) and patients with DSM-IV diagnosis of MDD (*n* = 61) with (*n * = 22) and without (*n * = 39) a history of suicide attempt, 18–73 years of age (mean age: 36 ± 13 years, Table 1). The proportion of Caucasian subjects was significantly higher in the MDD group than among healthy volunteers; the groups were otherwise balanced.

### Fatty acid differences across diagnostic groups

We found pointwise significant differences between MDD and healthy volunteer subjects, with respect to PUFAs (Table S2 in Supplementary Material); however, none were significant after multiple testing correction (*k* = 25 tests, using Benjamini–Hochberg adjustment). For 18:3*n*-3 (ALA), 20:5*n*-3 (EPA), and 20:2*n*-6, the MDD group had lower levels on the 0.05 level as compared to controls. Numeric differences were similarly seen in ratios of certain longer- to shorter-chain PUFAs, providing indirect indices of activity of enzymes in the LC-PUFA biosynthetic pathway. The ratio 22:5*n*-3/20:5*n*-3, reflecting elongase (*Elovl5*) activity, was higher in MDD, as was the ratio 20:3*n*-6/20:2*n*-6, which reflects Δ8-desaturase activity (*Fads2*), whereas the ratio 22:6*n*-3/22:5*n*-3, which reflects Δ-6 desaturase activity but also includes elongase (*Elovl2*) and β-oxidation steps, was lower (see Figure 1, Table S2 in Supplementary Material). Within the MDD group comparing suicide attempters and non-attempters, differences in the 22:4*n*-6 and its ratio with 20:4*n*-6, reflecting elongase (*Elovl5*) activity, were detected but were no longer significant after adjustment for multiple testing. Interestingly, these were both higher in suicide attempters, and trend associations were seen in attempters with higher 22:5*n*-3 (DPA) levels and lower ratios of 20:3*n*-6/18:3*n*-6, also reflecting elongase (*Elovl5*) activity. Our study was powered to detect relatively large (Cohen’s *d* > 0.7) effect sizes for a diagnosis of MDD, and very large (*d* > 1) effect sizes for suicide attempt after adjustment for multiple testing; thus, larger or more focused studies are needed for confirmatory analysis of the risk associated with some of these biological measures. All comparisons were adjusted for age and sex; adjusting for ethnicity and race did not alter the significance levels.

### DNA methylation differences across diagnostic groups

Using methylation pyrosequencing, we assayed DNA methylation patterns across regulatory regions of *Fads1*, *Fads2*, and *Elovl5* genes (seven separate regions, see Figure S1 in Supplementary Material). Across the diagnostic groups, the MDD patients exhibited a trend toward lower CpG methylation within the *Fads2* upstream but higher methylation at the *Elovl5* upstream proximal regions from the TSS as compared to controls (Table 2 and Figure S1 in Supplementary Material). Within the MDD group, suicide attempters showed significantly lower CpG methylation levels within the downstream *Elovl5* TSS region (*p* = 0.0028 adjusted) but higher methylation in the upstream *Elovl5* region compared to MDD non-attempters (*p* = 0.0036 adjusted; Table 2). Adjusting for race and ethnicity did not alter the findings.

**Table 2:** **Methylation levels (log-transformed) by group**.

Methylationsites	Controls (*n * = 59)		MDD (*n * = 61)		Comparison (adj. for age and sex)			MDD non-attempters (*n * = 39)		MDD suicide attempters (*n * = 22)		Comparison (adj. for age and sex)		
	Mean	SD	Mean	SD	*t*-score	*p*	Adj. *p*	Mean	SD	Mean	SD	*t*-score	*p*	Adj. *p*
*Fads1*U	2.79	0.39	2.75	0.31	−0.50	0.620	0.620	2.76	0.37	2.71	0.16	−0.85	0.396	0.554
*Fads1*CpG	1.27	0.28	1.23	0.29	−0.62	0.535	0.620	1.24	0.29	1.20	0.28	−0.64	0.524	0.611
*Fads1*D	1.21	0.41	0.96	0.89	−2.01	0.046	0.107	1.02	0.73	0.84	1.14	−0.94	0.349	0.554
*Fads2*U	2.97	0.42	2.79	0.39	−2.54	0.012	**0.042**	2.79	0.44	2.80	0.27	−0.35	0.726	0.726
*Fads2*D	4.21	0.19	4.27	0.16	1.56	0.121	0.212	4.24	0.18	4.31	0.08	0.97	0.335	0.554
*Elovl*U	2.26	0.57	2.53	0.58	2.54	0.012	**0.042**	2.36	0.48	2.85	0.62	3.29	**0.002**	**0.007**
*Elovl*D	1.88	0.26	1.84	0.25	−0.64	0.526	0.620	1.91	0.26	1.70	0.19	−3.74	**<0.001**	**0.003**

*“U” and “D” refer to upstream and downstream sites, respectively. MDD, major depressive disorder. Significant *p*-values after adjustment for multiple testing at the FDR = 0.05 level are shown in bold*.

### Contribution of fatty acid and DNA methylation signatures to diagnostic outcome

Spearman correlation coefficients computed between PUFA and DNA methylation measures indicate that there are some strong associations between measures in the two groups. 25 out of the 175 pairs (14%) were significantly correlated after adjustment for multiple testing at the FDR = 5% level (data not shown). In an effort to understand the relative contributions of PUFA and methylation patterns to disease risk with respect to MDD and to suicidal behavior, we performed model selection using LASSO logistic regression, starting with the full set of PUFA and methylation variables (including age, race, ethnicity, and sex, resulting in 36 variables), with no pre-screening, and using cross-validation to select the optimal model. This technique shrinks the coefficients in a (logistic) regression model until some estimates become effectively 0, meaning that the variable is no longer in the model. No variables were selected for the final model with MDD diagnosis as outcome. For suicide attempt risk within the MDD subjects, only two biological measures remained in the final model: CpG methylation at the *Elovl5* upstream and downstream regions. Gain and loss of methylation at the *Elovl5* upstream and downstream regions, respectively, independently contributed to suicide attempt risk (LASSO model with shrunk coefficients: upstream *Elovl5* methylation OR = 1.07, downstream *Elovl5* methylation OR = 0.89; logistic regression model results for the same variables: upstream: OR = 1.10, *z * = 2.33, *p * = 0.020; downstream: OR = 0.34, *z * = −2.96, *p * = 0.003, see Table 3). Age was also included in the final model for suicide attempt (LASSO model OR = 0.98; logistic model OR = 0.93, *z * = −2.05. *p * = 0.040).

**Table 3:** **Logistic regression model with suicide attempt as outcome in MDD patients**.

Predictors^a^	Odds ratio	95% CI		*Z*-statistic	*P*-value
		Lower	Upper	
Age	0.93	0.86	0.997	−2.05	0.040
*Elovl* U	1.10	1.02	1.21	2.33	0.020
*Elovl* D	0.34	0.17	0.70	−2.96	0.003

*Variables for the model were selected with cross-validated LASSO logistic regression*.

*^a^“U” signifies upstream, “D,” downstream regions*.

## Discussion

We found that DNA methylation of genes involved in *n*-3 PUFA biosynthesis was associated with MDD and suicide risk. PUFA imbalances long-term could induce changes in the epigenome and the methylome, which our data show are associated with suicide. There is ample precedent for PUFA effects on DNA methylation states (25, 27, 39), which have been attributed to effects of DHA on one-carbon metabolism (40). In this schema (41), DHA-containing phosphatidyl ethanolamine (PE) is a methyl acceptor, converted thereby to PC for PUFA transport through the bloodstream; when DHA is in short supply, it is hypothesized that methyl groups then may be available for other transmethylation of DNA or histones. Two out of seven methylation measures but no PUFA measures (out of a total of 25) made it into the multi-predictor model of suicide attempt risk; thus, methylation effect sizes were greater than those of PUFAs. Studies with larger sample sizes and/or focused on fewer comparisons may identify significant PUFA correlates of suicide attempt risk. However, it is also possible that the risk effects associated with PUFA levels were explained by methylation states, which may serve as stable proxies of PUFA composition.

Among MDD patients, we found that *Elovl5* methylation in suicide attempters was higher in the upstream region and lower in the downstream region, identifying potential epigenetic markers of suicide risk and suggesting that methylation in these two shore regions might have opposite functional effects. The effects of elongase activity on the generation of LC-PUFA are complex, due to competition between *n*-3 and *n*-6 PUFAs for the catalytic sites, the occurrence of genetic polymorphisms such as rs2397142, which confers low *Elovl5* activity (42), and the contribution of dietary intake.

Polyunsaturated fatty acid balance has therapeutic implications for treating chronic psychiatric disease, including MDD, bipolar disorder, and suicide risk. Randomized clinical trials have found *n*-3 PUFA supplements rich in eicosapentaenoic acid (EPA, 20:5*n*-3) to be effective in major depression (13, 43–45), although some concern about publication bias exists (13). It is also noteworthy that many mood stabilizing drugs (valproate, lithium, carbamazepine, olanzapine, and clozapine) specifically inhibit membrane turnover and downstream signaling of *n*-6 PUFAs while some (fluoxetine and imipramine) but not all (bupropion) antidepressants tested cause increased *n*-6 PUFA turnover (46).

To fit models for MDD diagnosis and suicide attempt based on all PUFA and methylation variables would require a sample size that is beyond the scope of the current study. Stepwise variable selection is a possible approach; however, the resulting model may be unstable and significance levels unreliable. We elected to use the LASSO method (in a logistic regression setting) and present the resulting parsimonious model for suicide attempt risk involving the two *Elovl5* methylation measures and age, with the odds ratios estimated by the shrinkage method LASSO employs. We also present in Table 3 the logistic regression model based on the same three predictors, estimated the classical way; the LASSO estimates are noticeably smaller, toward the neutral value (OR = 1). Since the LASSO model was selected using cross-validation, the odds ratios reported by it are likely to be more realistic for predicting outcome in future subjects. No variable was selected for predicting MDD, perhaps indicating that the associations between the biological variables and MDD diagnosis were not strong enough in the present sample after cross-validation.

This study has a number of limitations. As a cross-sectional study, it was not possible to determine how differences in DNA methylation and PUFA composition are associated with suicide risk long-term. Some of the results presented did not survive correction for multiple comparisons, possibly due to limited power, and therefore future studies should investigate DNA methylation and PUFA changes in a larger cohort. We did not examine relationships between epigenetic changes and the presence of genetic polymorphisms, which have been shown to impact PUFA levels [reviewed in Ref. (47)]. We investigated DNA methylation changes within regulatory regions of genes involved in PUFA biosynthesis, but it is likely that imbalances in dietary PUFA levels will induce DNA methylation modifications on a genome-scale not examined in this study. The lack of RNA specimens for the samples used in the present study did not allow for assessments of the relationship between changes in DNA methylation and corresponding gene expression. Still, compared to RNA, DNA methylation patterns are highly stable long-term, potentially serving as useful markers of dietary intake and deficiencies.

## Conclusion and Future Directions

These findings are intriguing because they establish the link between imbalances in PUFA composition and DNA methylation patterns in regulatory regions of genes directly involved in PUFA biosynthesis using a clinical cohort well-characterized with respect to depression and history of suicidal behavior. Investigations of epigenetics and dietary PUFAs can inform our understanding of gene–diet interactions and their relationship to psychiatric disease susceptibility and specifically, suicide risk. Future prospective studies of PUFA supplementation that include epigenetic assessments could open up a novel approach to personalized treatment of depression and suicidal behavior through modulation of disease-associated epigenetic marks by nutritional intervention.

## Conflict of Interest Statement

The authors declare that the research was conducted in the absence of any commercial or financial relationships that could be construed as a potential conflict of interest.

## Supplementary Material

The Supplementary Material for this article can be found online at http://journal.frontiersin.org/article/10.3389/fneur.2015.00092
