# Supplementary material for: DNA Methylation Perturbations in Genes Involved in Polyunsaturated Fatty Acid Biosynthesis Associated with Depression and Suicide Risk
Source: Front Neurol. 2015 Apr 28;6:92. doi: 10.3389/fneur.2015.00092 (PMC4412056; doi:10.3389/fneur.2015.00092)
Supplement: Supplementary file 1 [file Data_Sheet_1.DOCX]

**Supplementary Materials**

| **Gene** | **Primer** | | | **Sequence** | **Amplicon Size**  **(bp)** | **Region Amplified**  **(GRCh37/hg19)** | **Region Sequenced**  **(GRCh37/hg19)** | **Proximity to TSS**  **(bp)** |
| --- | --- | --- | --- | --- | --- | --- | --- | --- |
| ***Fads1* Upstream** | | F | TGGTTTTATTTTTTAGGGTTATGAGAT | | 238 | chr11:61582320-61582557 | chr11:61582428-61582504 | -161 to -85 |
|  | | R | ACACAACCCAAAATAACTACCTTAA | |  |  |  |  |
|  | | S | TTTAAAAATTAAGAAGAGGAAGAT | |  |  |  |  |
| ***Fads1* Downstream** | | F | AGAGGTAAATAGGGTTATAAAAATTGTG | | 220 | chr11:61584780-61584999 | chr11:61584788-61584848 | +2199 to +2259 |
|  | | R | CCTCCAAAATTAAAAACTACTACCTAC | |  |  |  |  |
|  | | S | ATTGTAATTTTTAAGGGTTTTTAG | |  |  |  |  |
| ***Fads1***  **CpG Island** | | F | GGAGATGGAGTAGGAAAGGAGTTTAT | | 175 | chr11:61583374-61583839 | chr11:61583423-61583502 | +834 to +913 |
|  | | R | ATACCATTCCCAATCCCTTATA | |  |  |  |  |
|  | | S | AGGATGGGGTTGAAA | |  |  |  |  |
| ***Fads2* Upstream** | | F | AGTAGGTTTTGGATTAGAGGGTTTTGA | | 152 | chr11:61594818-61594969 | chr11:61594841-61594939 | -872 to -744 |
|  | | R | AATTCCTCATCCCCCCCTTCCTCT | |  |  |  |  |
|  | | S | TTTTAGTTAGATAATTTTAGAAAAG | |  |  |  |  |
| ***Fads2* Downstream** | | F | GTTGTGGGTTTTTTTGGAGTTATTG | | 257 | chr11:61596909-61597165 | chr11:61596951-61597021 | +1238 to +1308 |
|  | | R | ATTTCTATATCCCATCAAACCTTTTTTCTC | |  |  |  |  |
|  | | S | AGTTATTGTTGTTTATTTTATTTTT | |  |  |  |  |
| ***Elovl5* Upstream** | | F | TGGTTGAGAAGTGTAAGTAAAATAAGTGT | | 345 | chr6:53212273-53212617 | chr6:53212313-53212412 | -1412 to -1313 |
|  | | R | CCCCATCCACAATTATAAATTAAACTC | |  |  |  |  |
|  | | S | AGTAAAATAAGTGTAGATTAATAGG | |  |  |  |  |
| ***Elovl5* Downstream** | | F | GAGTTTAGGTAGGGGTTGGTTT | | 238 | chr6:53213924-53214161 | chr6:53214042-53214121 | +317 to +396 |
|  | | R | CCCCTTCAACATAACATAACTCATA | |  |  |  |  |
|  | | S | GTAATAGTTTAGGTGAAGTTGT | |  |  |  |  |

**Table S1:** Primer sequences used for PCR and Pyrosequencing. Primers used for amplification of bi-sulfite DNA are denoted as F and R, while the sequencing primer used to assay the CpG sites of interest is denoted as S. Regions of interest were chosen using the ENCODE project’s hg19 construct. The proximity of the assayed sequence relative to the Transcription Start Site (TSS) and whether the region of interest was upstream (-) or downstream (+) of the TSS are also provided.

| **PUFA species (log-transformed)** | **Controls**  **(*n*=59)** | | **MDD**  **(*n*=61)** | | **Comparison**  **(Adjusted for age and sex)** | | **MDD nonattempters**  **(*n*=39)** | | **MDD suicide attempters**  **(*n*=22)** | | **Comparison**  **(Adjusted for age and sex)** | |
| --- | --- | --- | --- | --- | --- | --- | --- | --- | --- | --- | --- | --- |
|  | *Mean* | *SD* | *Mean* | *SD* | *t-score* | *P* | *Mean* | *SD* | *Mean* | *SD* | *t-score* | *P* |
| 18:2n-6 | 6.89 | 0.22 | 6.86 | 0.20 | -0.62 | 0.5372 | 6.87 | 0.22 | 6.84 | 0.18 | -0.37 | 0.7158 |
| 18:3n-6 | 2.61 | 0.55 | 2.61 | 0.57 | 0.14 | 0.8854 | 2.58 | 0.54 | 2.66 | 0.63 | 1.21 | 0.2332 |
| 20:2n-6 | 1.74 | 0.39 | 1.56 | 0.38 | -2.59 | 0.0107 | 1.62 | 0.35 | 1.45 | 0.40 | -1.64 | 0.1065 |
| 20:3n-6 | 3.82 | 0.38 | 3.79 | 0.36 | -0.25 | 0.8011 | 3.82 | 0.36 | 3.74 | 0.36 | -0.29 | 0.7724 |
| 20:4n-6 | 5.56 | 0.27 | 5.47 | 0.26 | -1.61 | 0.1093 | 5.48 | 0.26 | 5.45 | 0.25 | 0.15 | 0.8802 |
| 22:4n-6 | 2.00 | 0.37 | 1.97 | 0.37 | -0.37 | 0.7105 | 1.91 | 0.35 | 2.06 | 0.39 | 2.03 | 0.0475 |
| 22:5n-6 | 2.10 | 0.62 | 1.93 | 0.42 | -1.58 | 0.1161 | 1.88 | 0.46 | 2.03 | 0.33 | 1.43 | 0.1578 |
| 18:3n-3 | 3.08 | 0.39 | 2.91 | 0.42 | -2.21 | 0.0288 | 2.94 | 0.42 | 2.86 | 0.42 | -0.48 | 0.6335 |
| 20:5n-3 | 3.12 | 0.59 | 2.84 | 0.56 | -2.51 | 0.0133 | 2.85 | 0.61 | 2.82 | 0.47 | 0.64 | 0.5247 |
| 22:5n-3 | 2.40 | 0.60 | 2.49 | 0.53 | 0.93 | 0.3550 | 2.44 | 0.56 | 2.59 | 0.46 | 1.76 | 0.0836 |
| 22:6n-3 | 4.04 | 0.44 | 3.89 | 0.46 | -1.60 | 0.1114 | 3.91 | 0.52 | 3.87 | 0.33 | 0.03 | 0.9776 |
| 18:3n-6/18:2n-6 | -4.28 | 0.58 | -4.25 | 0.52 | 0.39 | 0.6958 | -4.30 | 0.49 | -4.18 | 0.58 | 1.46 | 0.1486 |
| 20:3n-6/18:3n-6 | 1.21 | 0.48 | 1.18 | 0.39 | -0.42 | 0.6729 | 1.24 | 0.38 | 1.08 | 0.41 | -1.96 | 0.0547 |
| 20:3n-6/20:2n-6 | 2.08 | 0.42 | 2.23 | 0.38 | 2.33 | 0.0218 | 2.20 | 0.33 | 2.29 | 0.46 | 1.44 | 0.1558 |
| 20:4n-6/20:3n-6 | 1.73 | 0.35 | 1.68 | 0.28 | -1.01 | 0.3128 | 1.66 | 0.28 | 1.71 | 0.30 | 0.44 | 0.6632 |
| 20:4n-6/18:2n-6 | -1.33 | 0.30 | -1.39 | 0.24 | -1.01 | 0.3130 | -1.39 | 0.25 | -1.38 | 0.23 | 0.46 | 0.6468 |
| 22:4n-6/20:4n-6 | -3.56 | 0.28 | -3.50 | 0.29 | 0.96 | 0.3372 | -3.57 | 0.26 | -3.39 | 0.30 | 2.49 | 0.0159 |
| 22:5n-6/22:4n-6 | 0.10 | 0.58 | -0.03 | 0.44 | -1.34 | 0.1827 | -0.03 | 0.48 | -0.03 | 0.39 | -0.16 | 0.8755 |
| 22:5n-6/18:2n-6 | -4.79 | 0.68 | -4.93 | 0.42 | -1.25 | 0.2138 | -4.99 | 0.44 | -4.81 | 0.35 | 1.64 | 0.1071 |
| 22:5n-6/20:4n-6 | -3.46 | 0.61 | -3.54 | 0.43 | -0.80 | 0.4242 | -3.60 | 0.46 | -3.42 | 0.37 | 1.33 | 0.1873 |
| 20:5n-3/18:3n-3 | 0.03 | 0.59 | -0.07 | 0.51 | -0.89 | 0.3772 | -0.09 | 0.52 | -0.04 | 0.49 | 1.10 | 0.2763 |
| 22:5n-3/20:5n-3 | -0.72 | 0.78 | -0.34 | 0.54 | 2.89 | 0.0045 | -0.41 | 0.58 | -0.24 | 0.44 | 1.01 | 0.3158 |
| 22:6n-3/22:5n-3 | 1.64 | 0.60 | 1.40 | 0.55 | -2.19 | 0.0305 | 1.46 | 0.57 | 1.28 | 0.50 | -1.56 | 0.1237 |
| 22:6n-3/18:3n-3 | 0.96 | 0.49 | 0.98 | 0.50 | 0.39 | 0.6970 | 0.97 | 0.49 | 1.01 | 0.52 | 0.43 | 0.6685 |
| 22:6n-3/20:5n-3 | 0.92 | 0.44 | 1.06 | 0.44 | 1.60 | 0.1119 | 1.06 | 0.44 | 1.05 | 0.45 | -0.75 | 0.4587 |

**Table S2.** Plasma PUFA levels and plasma ratios of PUFA product to substrate compared by diagnostic group and suicide attempt status. No significant associations were found after adjustment for multiple testing.

| **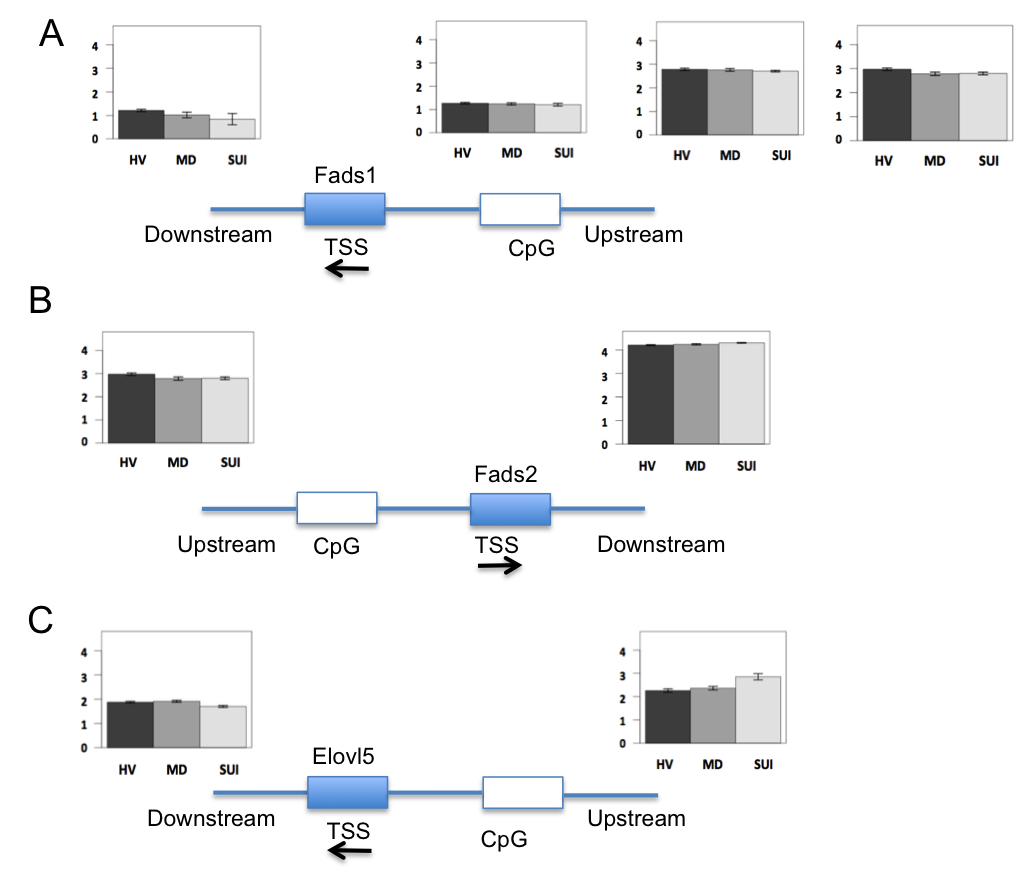** |
| --- |
| **Figure S1**. DNA methylation differences in (A) *Fads1*, (B) *Fads2*, and (C) *Elovl5* promoter associated regions among MDD suicide attempters, MDD non-attempters, and controls. Bars represent DNA methylation levels averaged across individual CpG sites for healthy volunteers (HV, dark gray), MDD non-attempters (MD, light gray), and MDD suicide attempters (SUI, white).  Error bars representing the standard error of the mean are also included. |

.
